# Data-efficient image transformer for landscape character classification and visual comfort prediction in Chinese hospitals

**DOI:** 10.3389/frai.2026.1733709

**Published:** 2026-05-08

**Authors:** Yue Li, Guo Yue, Riyadh Mundher, Abdulrahman M. Abdulghani

**Affiliations:** 1 School of Art and Design, Shandong Women’s University, Ji’nan, China; 2 School of Architecture and Design, Jiangxi University of Science and Technology, Ganzhou, Jiangxi, China; 3 Department of Landscape Architecture, Faculty of Design and Architecture, Universiti Putra Malaysia, Serdang, Malaysia; 4 Department of Communication Technology and Network, Faculty of Computer Science and Information Technology, Universiti Putra Malaysia, Serdang, Malaysia

**Keywords:** attention mechanism, data-efficient image transformer, hospital landscape, landscape character, therapeutic landscape, vision transformer, visual comfort

## Abstract

In hospital landscapes, where visual comfort influences stress recovery and patient satisfaction, reliable computational tools are needed to link landscape character with human perception. However, existing research on therapeutic landscapes in healthcare has largely focused on qualitative evaluations and design guidelines, with limited development of integrated, interpretable computational models that quantitatively connect landscape characteristics with human perception outcomes. This study addresses this gap by developing an AI-driven decision support system that integrates landscape character classification with visual comfort prediction in Chinese hospital settings. From 488 images collected across three hospitals, 30 representative images were evaluated by 252 respondents. Perception scores were assigned to all images based on landscape character, creating a labeled dataset. A Data-Efficient Image Transformer (DeiT) with dual prediction heads was developed for simultaneous landscape character classification and continuous visual comfort score regression. The model achieved a 96.34% classification accuracy and a mean absolute error (MAE) of 0.055 for visual comfort prediction, substantially outperforming ResNet-50 (accuracy: 89.39%; MAE: 0.148) and the standard Vision Transformer (ViT) (accuracy: 94.06%; MAE: 0.155). The DeiT model demonstrated 19–26% faster convergence and 62–65% improved visual comfort prediction. These results demonstrate that therapeutic landscape qualities, often regarded as subjective, exhibit consistent, computationally learnable patterns. The validated framework provides landscape architects, hospital planners, and administrators with an evidence-based tool for systematic therapeutic landscape evaluation and optimization.

## 1 Introduction

### 1.1 Landscape character and human visual perception

Landscape character is described as “a distinct and recognizable set of landscape elements that makes one landscape different from another, rather than better or worse” (Mundher et al., 2022). The term refers to the distinctive, familiar pattern of elements that recur in a particular landscape type and together shape landscape identity. The European Landscape Convention defines landscape character as “a recognizable spatial pattern influenced by ecological and cultural processes,” underscoring the need for integrated approaches that capture both natural and anthropogenic dynamics (Karim et al., 2023). Landscape character identification is fundamental for planning, conservation, and sustainable management; however, traditional approaches such as field surveys, manual geographic information system GIS delineation, and expert interpretation are often limited by subjectivity and scalability constraints (Huang et al., 2025). In urban contexts, landscape character classifications have been linked to positive emotions and health benefits, informing the design of therapeutic green spaces (Dmitrović et al., 2025). In China, a methodology has been applied to characterize landscape images by identifying landscape characters based on physical and visual attributes (Zhao et al., 2023). Similarly, in Vietnam, landscapes were assessed using a definitional framework that integrates physical and visual variables to classify landscape character types (Wang et al., 2023). Overall, landscape character plays a vital role in conservation, development, and urban planning, providing a framework for understanding and managing landscapes sustainably.

Human visual perception is a multifaceted process that involves decomposing scenes into objects and integrating influences beyond raw sensory input (Peters and Kriegeskorte, 2021). Also, the high-level properties of visual perception of different social groups can be captured through the coding of interview or questionnaire data (Jiang et al., 2025). Perception theories indicate that landscape character strongly influences visual perception, suggesting that people evaluate landscapes based on visual stimuli (Gülçin and Yalçınkaya, 2024). Urban environments illustrate this relationship: visual qualities such as openness or broadness and complexity or crowding shape human perception (Kronemer et al., 2022). Landscape elements and density likewise play important roles and are associated with visually pleasing and comfortable experiences (Farahani et al., 2023). Together, these studies confirm the link between landscape character and human visual perception, underscoring the need for integrated approaches to improve visual quality and human experience. Additionally, incorporating diverse stakeholders’ visual perceptions and recent advances in AI-driven landscape management helps ensure inclusive decision-making and secure sustainable support (Li et al., 2024).

### 1.2 Recent advances in AI-driven landscape evaluation

In recent years, the use of artificial intelligence (AI) to overcome constraints in the study of landscape character and human visual perception has increased (Yang et al., 2025). Studies have shown that machine learning methods can model urban landscape patterns, assess changes in protected areas, and classify land cover with greater accuracy and adaptability than that achieved by manual or conventional statistical techniques (Gremes et al., 2024; Huang et al., 2025). For example, combining vegetation indices with deep learning architectures has improved the detection of cropland abandonment, offering insights into food security and environmental degradation (Karim et al., 2023). Likewise, lightweight transformer-based models have been optimized for land-cover classification, delivering higher efficiency while reducing computational costs, which is crucial for edge-computing applications such as drone-based surveys (Rozario et al., 2024). Related advances have extended into healthcare, where hybrid deep-learning frameworks integrated with Internet of Things (IoT) devices have achieved high accuracy in real-time disease detection (Abdulghani et al., 2023). Although distinct from landscape monitoring, these studies underscore the versatility of deep learning and multimodal data integration in domains where precision and reliability are critical (Rozario et al., 2024; Xing et al., 2025).

In parallel, AI applications in landscape architecture have moved beyond classification and monitoring to include design, management, and user interaction (Cai, 2024). Intelligent systems are now employed for automated design generation, optimization of spatial layouts, ecological simulations, and maintenance strategies such as smart irrigation and pest detection (Xing et al., 2025). These applications highlight AI’s potential to respond to pressures of urbanization, ecological degradation, and management inefficiencies. AI has also been applied to evaluate the aesthetic and perceptual qualities of natural environments (Mundher et al., 2023a). Predictive models based on neural networks, particularly radial basis function models, have outperformed traditional regression methods in forecasting visual landscape quality, offering decision-support tools for landscape management (Jahani and Rayegani, 2020). Such approaches integrate quantitative environmental parameters with perception-based assessments, bridging scientific analysis and human experience. However, researchers stress that AI should complement rather than replace human expertise since professional judgment, aesthetics, and the cultural context remain essential to decision-making (Jahani and Rayegani, 2020; Xing et al., 2025).

One of the most significant recent advances in AI is the Vision Transformer (ViT), which marks a substantive methodological shift. ViTs extend transformer architectures from natural language processing to computer vision, offering a competitive alternative to convolutional neural networks (CNNs) (Heo et al., 2021; Liu and Aldrich, 2024). By employing self-attention to model global spatial dependencies, ViTs excel at tasks requiring global feature extraction, such as image recognition and restoration, and have shown superior performance in complex classification problems (Abdulghani et al., 2023; Ali et al., 2023; Rozario et al., 2024). Enhancements such as Mobile ViT architectures integrate convolutional layers with attention mechanisms to reduce parameter counts without sacrificing accuracy, making them suitable for resource-constrained environments (Rozario et al., 2024). The integration of multimodal approaches, combining ViTs with textual or linguistic data, further extends landscape analysis to include descriptive and qualitative inputs, providing more nuanced characterizations (Huang et al., 2025; Xing et al., 2025). The ability of ViTs to capture global spatial relationships parallels how humans perceive therapeutic qualities through the holistic composition of natural and built elements, making them well suited for modeling the subtle visual qualities that contribute to healing environments (Bazi et al., 2021). Overall, the literature shows a shift from manual, subjective landscape character identification methods to advanced, multimodal, AI-driven frameworks. The convergence of ViTs, natural language processing (NLP), and GIS enables scalable and interpretable solutions for land cover classification, cropland monitoring, and aesthetic evaluation (Jahani and Rayegani, 2020; Rozario et al., 2024; Xing et al., 2025). Future research should prioritize explainable AI to enhance transparency and trust, integrate high-quality multimodal datasets, and balance computational efficiency with ecological and cultural sensitivity (Xing et al., 2025).

### 1.3 Research problem and aim

Assessing the quality of the therapeutic landscape in healthcare environments presents a fundamental methodological challenge. Traditional evaluation approaches rely on expert judgment through post-occupancy evaluations or visual preference surveys (Al-Sharaa et al., 2022; Belčáková et al., 2018; Mundher et al., 2023b), methods that, while valuable for capturing nuanced qualitative insights, face critical limitations when applied at scale across healthcare systems. A hospital planning authority cannot feasibly conduct comprehensive expert assessments of every outdoor space, pathway, courtyard, and garden view encountered by patients, visitors, and staff (Al-Sharaa et al., 2022; Bernhardt et al., 2022; Totaforti, 2018). This scalability constraint becomes particularly acute in rapidly expanding healthcare systems such as those in China, where hundreds of new hospitals are constructed annually, each containing dozens to hundreds of distinct landscape views requiring therapeutic optimization (Belčáková et al., 2018; Totaforti, 2018).

Therapeutic landscape assessment involves simultaneously recognizing and categorizing landscape types based on compositional characteristics while also evaluating potential therapeutic quality through factors such as spatial openness, vegetation density, water presence, and built–natural element integration (Mundher et al., 2023a; Mundher et al., 2022). Existing frameworks typically address these tasks independently: classification systems focus on landscape typology (Wang et al., 2023; Zhao et al., 2023), whereas quality assessment tools emphasize preference or uplift scoring (Jahani and Rayegani, 2020; Mundher et al., 2023b), failing to model their intrinsic relationships. Furthermore, studies comparing landscape evaluations document inter-rater agreement coefficients of 0.80–0.88 among trained professionals (Mundher et al., 2023a), indicating 12–20% variability that complicates evidence-based decision-making when administrators require consistent metrics to prioritize landscape investments.

Recent advances in ViT architectures present opportunities to address these limitations through systematic, scalable computational assessment (Heo et al., 2021; Huang et al., 2025; Rozario et al., 2024). Unlike convolutional approaches, transformers model global spatial relationships through self-attention mechanisms that parallel how humans perceive therapeutic qualities through holistic landscape composition (Jahani and Rayegani, 2020; Xing et al., 2025). However, standard ViTs require large, labeled datasets of thousands to millions of images for effective training (Heo et al., 2021), creating a fundamental data scarcity challenge in therapeutic landscape domains where each image requires expensive expert evaluation or large-scale perception surveys. Data-efficient Image Transformers (DeiTs) address this constraint through knowledge distillation mechanisms enabling learning from smaller datasets (Wang et al., 2025), making them particularly suitable for specialized applications such as therapeutic landscape assessment where labeled data acquisition is prohibitively expensive.

This study addressed these converging challenges by developing an attention-based visual analysis framework for therapeutic landscape assessment within a unified DeiT model-based architecture. Focusing specifically on Chinese hospital landscapes, a critical yet underexplored context, the framework integrates systematic landscape character taxonomy with large-scale human perception data to train an interpretable AI model. Thus, this study developed an AI-driven decision support system that integrates landscape character classification with human comfort perception data in Chinese hospital settings.

## 2 Materials and methods

**Figure 1:**
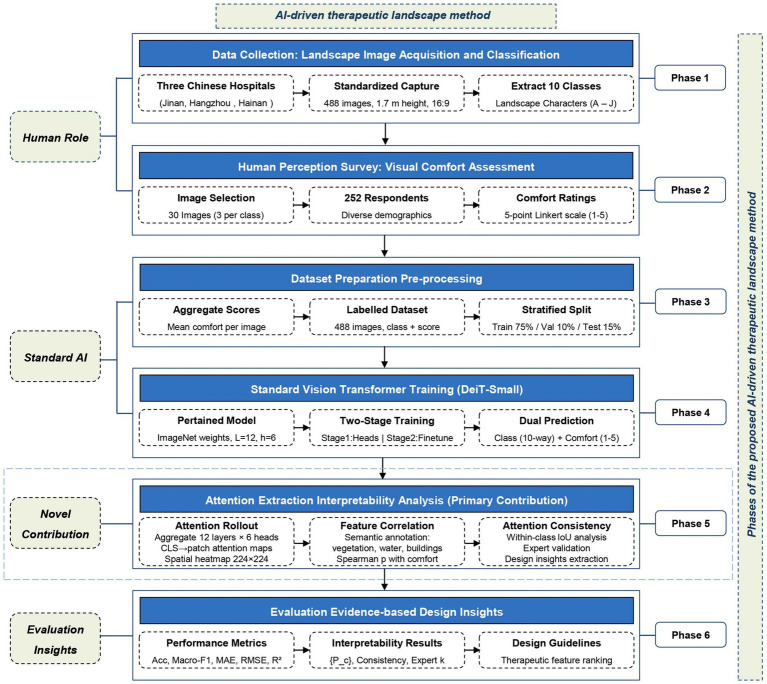
Phases of the proposed AI-driven therapeutic landscape method.

In this study, a hybrid approach that combines artificial intelligence with visual comfort assessment was adopted. The approach was grounded in landscape character and human visual perception in Chinese hospital settings. It integrates an image-acquisition protocol, landscape character classification, visual perception survey, and AI-driven visual comfort assessment to evaluate the therapeutic landscape ViT. Figure 1 illustrates the phases of the proposed method.

### 2.1 Study area

**Figure 2:**
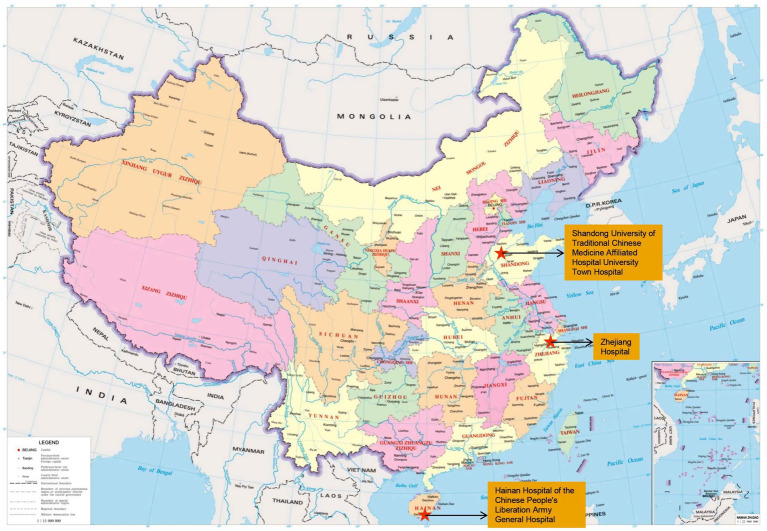
Study hospital locations in China.

There is growing recognition of the importance of incorporating visual comfort and therapeutic landscapes into hospital design to enhance patient recovery and wellbeing (Al-Sharaa et al., 2022). Therapeutic landscape design in hospitals, which involves incorporating natural elements and therapeutic environments, has been shown to improve recovery and provide relaxation spaces for patients (Belčáková et al., 2018). Biophilic design, which reconnects individuals with nature to improve their psychological and physical health, has also been shown to enhance wellbeing (Totaforti, 2018). This approach has been replicated in many hospitals, with therapeutic landscapes provided in psychiatric facilities to promote recovery (Simonsen and Duff, 2020). It also highlights the importance of green spaces in stroke care, as they help reduce stress and improve patient outcomes (Bernhardt et al., 2022). Overall, incorporating therapeutic landscapes into hospital design aims to create environments that facilitate healing and enhance the quality of care through the visual comfort of natural landscapes. In this study, three hospitals in China were selected: University Town Hospital in Jinan, Zhejiang Hospital in Hangzhou, and Hainan Hospital of the Chinese People’s Liberation Army (PLA) General Hospital in Hainan. Each hospital is distinguished by their provision of green spaces to support patients’ recovery and visual comfort (Figure 2).

- University Town Hospital, the Affiliated Hospital of Shandong University of Traditional Chinese Medicine, is located in Jinan, Shandong Province (36.65° N, 117.01° E), situated within a temperate continental monsoon climate zone characterised by mean summer temperatures of 26–30 °C, average relative humidity of 60–70%, and daily sunshine duration exceeding 8 h in May. Founded in 1946, it is a comprehensive Grade II-A hospital that integrates medical care, research, education, emergency services, health preservation, and rehabilitation. The overall architectural design follows a courtyard-and-garden concept, integrating the character of the university town’s mountain and water landscapes, natural ecology, green livability, and technological and cultural elements. Jinan is located in a temperate monsoon climate zone characterized by hot, humid summers and cold winters, with distinct seasonal variation in daylight and vegetation conditions, which influence outdoor visual perception and environmental comfort.
- Zhejiang Hospital is located in Hangzhou (30.27° N, 120.15° E), within a subtropical monsoon climate zone with mean summer temperatures of 28–33 °C and moderate humidity (relative humidity approximately 75%). Founded in 1954, it is a Grade III-A comprehensive hospital integrating medical treatment, teaching, scientific research, prevention, and health care, with a medical model characterized by multidisciplinary collaboration. The hospital also features extensive green spaces that aid patient recovery. Hangzhou has a humid subtropical climate with high humidity, abundant rainfall, and relatively long daylight hours in summer, which affect outdoor lighting conditions, vegetation growth, and visual landscape characteristics.
- Hainan Hospital of the Chinese PLA General Hospital is located in Sanya, Hainan (18.30° N, 109.73° E), within a tropical maritime climate zone characterised by consistently high solar radiation, mean summer temperatures of 29–34 °C, and daily sunshine duration exceeding 7 h. Founded in 2012 as a modern Grade III-A comprehensive hospital integrating medical treatment, health care, education, and research functions. Sanya has a tropical climate characterized by high temperatures, strong solar radiation, high humidity, and relatively stable daylight duration throughout the year. The differences in climate, daylight availability, solar radiation, vegetation conditions, and environmental characteristics among these three locations provide diverse visual environmental conditions for landscape perception studies, as environmental factors such as daylight, sky conditions, and surrounding landscape elements significantly influence visual comfort, glare perception, and environmental evaluation in outdoor environments.

These three hospitals were selected to ensure representativeness across three key dimensions: climate, institutional type, and landscape design. The hospitals are located in different climatic zones: temperate monsoon (Jinan), humid subtropical (Hangzhou), and tropical (Sanya), which provide variation in vegetation, sunlight conditions, and seasonal landscape characteristics that influence visual perception. They also represent different institutional types and development periods, including a traditional Chinese medicine hospital, a comprehensive multidisciplinary hospital, and a modern large-scale hospital, reflecting different architectural styles and functional layouts. In addition, the hospitals feature distinct landscape design approaches, including courtyard gardens, urban green spaces, and tropical landscape environments. Together, these differences ensure that the dataset captures a representative range of hospital landscape conditions in China, improving the generalizability of the model.

### 2.2 Data collection

#### 2.2.1 Landscape image acquisition

**Figure 3:**
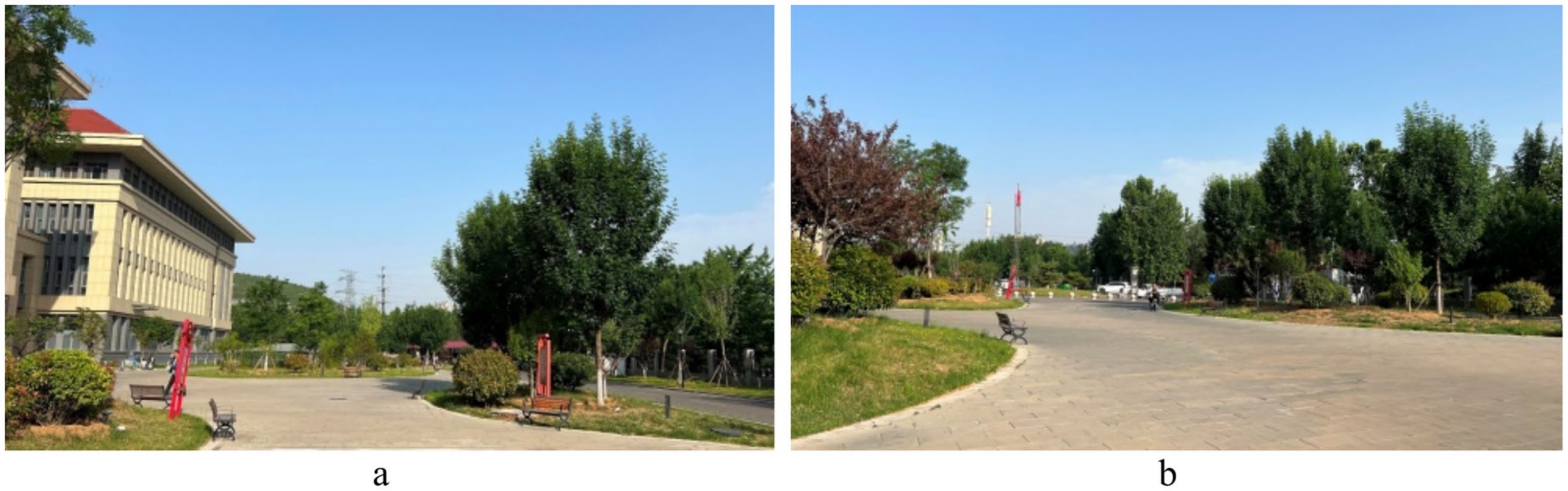
Examples of the images taken.

Standardization of image acquisition is critical to model reliability. All the images were captured in accordance with strict architectural photography principles to ensure consistency across the dataset. The camera height was fixed at 1.70 m, which is the average human eye level. Each image followed a standardized composition, with approximately two-thirds of the frame devoted to land elements and one-third to the sky, reflecting typical viewing patterns in outdoor hospital environments. Images were captured with a camera setting of (ISO 25, wide-angle 26 mm f/1.8 aperture, 9 MP), and a 16:9 landscape format was maintained throughout. All images were taken under consistent lighting, with no human presence, during the summer period (May 2025) to maintain temporal and seasonal consistency in vegetation appearance. All images were captured between 09:00 and 16:00 local time under clear-sky or lightly overcast natural daylight conditions to ensure uniform illumination intensity and minimize directional shadow effects (Figure 3).

#### 2.2.2 Landscape character classification

Images were captured along the circulation paths of the three hospitals following the standardized acquisition protocol. A total of 488 landscape images were systematically collected to capture the full diversity of therapeutic landscape configurations within each hospital. These images were grouped by similarity in view type (broad, enclosed, direct, crowded) and element type (vegetation, buildings, infrastructure, pathways, water), following the landscape character classification methodology of Gao et al. (2023). Landscape character groups represented by fewer than three images were treated as non-dominant, and those images were reassigned to the most similar dominant character group to ensure adequate representation. This classification process yielded 10 unique hospital landscape character groups (Groups A–J), each representing distinct combinations of spatial configuration and visual elements.

For the human perception survey, 30 representative images were strategically selected (three per landscape character group) to capture the characteristic features of each category while maintaining visual quality and clarity suitable for online presentation. These 30 images formed the basis of the perception assessment described in Section 2.3. Following the survey, the mean visual comfort scores for each landscape character group (Table 1) were assigned to all corresponding images within the full 488-image dataset. This score assignment strategy enabled supervised learning across the entire dataset while grounding predictions in empirical human perception data. The resulting labeled dataset of 488 images with both landscape character labels and visual comfort scores was partitioned for model training, validation, and testing as described in Section 2.4.2. Supplementary Table A presents all 30 survey images with their landscape character classifications. Table 2 details the compositional elements defining each landscape character group.

**Table 1:** Average mean scores of the landscape character groups.

Group	Landscape character	Code	Mean score	Average mean
E	Enclosed view of green vegetation with two-sided building	E01 E02 E03	3.08 3.01 3.29	3.13
F	Direct view of green vegetation with building in the background	F01 F02 F03	3.16 3.25 3.35	3.25
D	Enclosed view of green vegetation with one-sided building	D01 D02 D03	3.20 3.40 3.41	3.34
G	Direct view of green vegetation with infrastructure	G01 G02 G03	3.57 3.26 3.41	3.41
H	Crowded view of green vegetation with Infrastructure	H01 H02 H03	3.50 3.41 3.35	3.42
B	Crowded view of green vegetation	B01 B02 B03	3.57 3.22 3.71	3.50
A	Broad view of green vegetation	A01 A02 A03	3.80 3.35 3.46	3.54
I	Enclosed view of green vegetation with pathway	I01 I02 I03	3.46 3.58 3.70	3.58
C	Broad view of green vegetation with building	C01 C02 C03	3.53 3.61 3.72	3.62
J	Crowded view of green vegetation with water	J01 J02 J03	3.50 3.80 3.60	3.63

**Table 2:** Image classification by landscape character.

Group	Type of view				Type of element							Landscape character
	Broad View	enclosed view	Direct View	Crowded view	Green vegetation	Building*			Infrastructure	Pathway	Water	
						O	T	B				
A	x				x							Broad view of green vegetation
B				x	x							Crowded view of green vegetation
C	x				x			x				Broad view of green vegetation with building
D		x			x	x						Enclosed view of green vegetation with one-sided building
E		x			x		x					Enclosed view of green vegetation with two-sided building
F			x		x			x				Direct view of green vegetation with building in the background
G			x		x				x			Direct view of green vegetation with infrastructure
H				x	x				x			Crowded view of green vegetation with infrastructure
I		x			x					x		Enclosed view of green vegetation with pathway
J				x	x						x	Crowded view of green vegetation with water

*O, One-sided; T, Two-sided; B, Background-sided.

### 2.3 Human perception survey

The images (30 selected images) were randomized on an online survey platform, ensuring that no two images of the same landscape character group appeared consecutively to minimize response bias. The visual comfort survey comprised two sections: (A) demographics and (B) the image survey. Section (A) contained eight general questions, including gender, age, and educational level, as well as other study-relevant items. Section (B) presented the images and participants were asked, “Does this scene enhance visual comfort and therapeutic relaxation within the hospital setting?” Responses were given on a 5-point Likert scale (1 = strongly disagree, 5 = strongly agree). This single-question measure was selected for its directness, cognitive simplicity, and proven effectiveness in capturing holistic perceptual judgments in landscape assessment studies (Mundher et al., 2023b).

A total of 252 respondents completed the survey. Individual image scores were calculated as the mean rating across all respondents. Landscape character group scores were computed by averaging the three individual image scores within each landscape character group. These group-level mean scores were subsequently assigned to all images within the corresponding landscape character group in the full 488-image dataset, enabling comprehensive model training.

### 2.4 Attention-based visual analysis framework

An attention-guided computational framework (Zhao et al., 2024) was used to identify features associated with visual comfort in hospital landscape settings. Rather than proposing novel architectural contributions, established ViT attention mechanisms (Abdulghani et al., 2025a; Abdulghani et al., 2025b; Hamad et al., 2025) were leveraged to extract interpretable insights into landscape perception. This approach was used to determine which specific visual elements (vegetation density, water features, spatial openness, or architectural integration) most strongly influence perceived visual comfort and therapeutic value (Kang and Kim, 2019; Ma et al., 2025; Olszewska-Guizzo et al., 2022).

The framework was formulated as a dual-objective problem. Given a dataset D = {(xᵢ, gᵢ, sᵢ)}ᵢ=₁ᴺ of N standardized landscape images, where xᵢ ∈ ℝᴴˣᵂˣ^3^ represents an RGB image, gᵢ ∈ {A, B, …, J} denotes the landscape character group, and sᵢ ∈ [1, 5] represents the empirically measured visual comfort score from human surveys, two complementary objectives were pursued. First, a classification function f θ: X → G was learned to map images to landscape character groups, establishing baseline recognition capability (Yun, 2025). Second, and more critically for this study, attention weight distributions A ∈ ℝᴺˣᴺ were extracted and analyzed to identify which spatial regions and visual features correlate most strongly with visual comfort. This interpretability-focused approach distinguishes the present work from purely performance-oriented computer vision studies.

#### 2.4.1 Model architecture and selection rationale

A standard ViT architecture was employed for the DeiT-Small configuration (Wang et al., 2025). This architecture was selected not for novelty but for its well-documented interpretability properties. Unlike CNNs, which learn hierarchical features through spatial filtering, ViTs employ self-attention mechanisms that explicitly compute relationships between all image regions (Tian and Bai, 2024). These attention weights provide direct, quantifiable evidence of which landscape elements the model prioritizes when making visual comfort assessments, making them particularly suitable for design-oriented analysis Supplementary Table B, for mathematical notation used in the proposed model. The architecture processes input images x ∈ ℝ^224^ˣ^224^ˣ^3^ through the following pipeline:

- *Patch embedding*: Images were divided into non-overlapping patches of size P × *p* = 16 × 16 pixels, yielding *N* = (H × W)/P^2^ = 196 patches. Each patch was flattened and linearly projected to a D-dimensional embedding space, where D = 384 represents the model’s hidden dimension. A learnable classification token cls was prepended to the sequence, and positional encodings Epos ∈ ℝ^(^ᴺ^+1)^ˣᴰ were added to preserve spatial relationships, as shown in Equation 1.

$$z_{0}=[\mathrm{cls};E\cdot \;{\mathrm{xp}}_{₁};E\cdot \;{\mathrm{xp}}_{₂};\;\ldots ,E\cdot \;{\mathrm{xp}}_{n}]+\text{Epos}$$ (1)

Where E in $z_{0}$ ∈ ℝ^(^ᴾ^2•^^3)^ˣᴰ is the learnable patch projection matrix that linearly maps each flattened P x P x 3 patch to the D-dimensional embedding space, ${\mathrm{xp}}_{n}$ denotes the n-th flattened image patch, [cls] is the prepended learnable classification token whose final representation is used for downstream predictions, and Epos in ℝ ^((N + 1) x D)^ encodes spatial positional information to preserve the relative ordering of patches, which the self-attention mechanism would otherwise discard.

- *Transformer encoder*: The embedded sequence was processed through L = 12 identical transformer layers. Each layer l applied multi-head self-attention (MHSA) followed by a position-wise feed-forward network (FFN), with residual connections and layer normalization, as shown in Equations 2, 3. The multi-head attention mechanism computed attention weights across h = 6 parallel heads, each operating on a dk = D/h = 64-dimensional subspace, as shown in Equation 4.

$$z_{1}^{\prime }=\text{MHSA}(\mathrm{LN}(z_{1-1}))+z_{1-1}$$ (2)

$$z_{1}=\mathrm{FFN}(\mathrm{LN}(z_{1}^{\prime }))+z_{1}^{\prime }$$ (3)

$$\text{Attention}(Q,K,V)=\text{softmax}({\mathrm{QK}}^{T}/\surd \mathrm{dk})V$$ (4)

The scaling factor 1/sqrt(dk) prevents the dot-product Q*K^T^ from growing excessively large in magnitude as dk increases, which would push the softmax function into regions of very small gradients and impede learning. Here Q, K, and V are obtained by linearly projecting the input features using learned weight matrices WQ, WK, and WV, respectively. The attention matrix A = SoftMax (QKᵀ / √dk) ∈ ℝᴺˣᴺ provided interpretable weights indicating which image patches attend to which others during processing.

- *Task-specific prediction heads*: Two prediction heads operated on the final classification token representation zcls^(^ᴸ^)^. A classification head produced probability distributions over K = 10 landscape character classes, as shown in Equation 5. A regression head predicted continuous visual comfort scores ŝ ∈ [1, 5], as shown in Equation 6.

$$p=\text{softmax}(\mathrm{Wc}\;\cdot \;\mathrm{zcl}s^{\left(L\right)}+\mathrm{bc})$$ (5)

$$\hat{s}=4\;\cdot \;\sigma (\mathrm{Wr}\;\cdot \;\mathrm{zcl}s^{\left(L\right)}+\mathrm{br})+1$$ (6)

Where Wr and br are the learnable weight matrix and bias vector of the regression head, *σ* (.) denotes the element-wise sigmoid function that maps the linear output to the range (0, 1), and the scalar multiplier 4 combined with the additive bias +1 linearly rescales this output to the valid 5-point Likert scale range [1, 5], ensuring that all comfort predictions remain physiologically interpretable.

#### 2.4.2 Training protocol and regularization

Training was conducted using a transfer learning approach to leverage representations learned from large-scale natural image datasets while adapting to the domain-specific characteristics of therapeutic landscapes via the following steps:

- *Initialization*: Model weights were initialized from DeiT-Small pre-trained on ImageNet-1 K, providing robust feature extractors for natural scenes. This initialization strategy was motivated by prior work demonstrating that ImageNet pre-training transfers effectively to landscape-related tasks.
- *Data pre-processing*: A standardized pre-processing pipeline was applied to all the images, as shown in Equation 7.

$$x^{\prime }=\text{Normalize}(\text{Resize}(\text{Augment}(x)))$$ (7)

Here, the images were resized to 224 × 224 pixels and normalized using ImageNet channel-wise statistics (*μ* = [0.485, 0.456, 0.406], σ = [0.229, 0.224, 0.225]). During training, stochastic augmentation was applied: random horizontal flipping (*p* = 0.5), color jittering (brightness = 0.2, contrast = 0.2, saturation = 0.2, hue = 0.1), and random rotation (±15°). These transformations improved model robustness without introducing unrealistic landscape configurations.

- *Two-stage training strategy*: Training proceeded in two stages. Stage 1 (feature extraction): For T₁ = 20 epochs, the pre-trained transformer backbone was frozen, and only the task-specific prediction heads {Wc, bc, Wr, br} were optimized. A learning rate *η*₁ = 5 × 10^−4^ was employed with the AdamW optimizer, enabling rapid adaptation to landscape character categories while preserving general visual representations. Stage 2 (fine-tuning): For T₂ = 30 epochs, all model parameters *θ* were jointly optimized using a reduced learning rate *η*₂ = 5 × 10^−5^ with cosine annealing scheduling, as shown in Equation 8.

$$\eta_{t}=\eta \min+½(\eta \max–\eta \min)(1+\cos(\mathrm{t\pi}/T))$$ (8)

Where *t* is the index of the current training epoch (t = 0 at the start of Stage 2), T = T_2_ = 30 is the total number of fine-tuning epochs, *η*max = *η*^2^ = 5 × 10^−5^ is the initial learning rate at the start of fine-tuning, and *η*min = 1 × 10^−6^ is the minimum learning rate reached at epoch T. This schedule gradually reduces the learning rate following a cosine curve, allowing the model to make increasingly conservative parameter updates as it approaches convergence and reducing the risk of overshooting optimal weights.

- *Loss function*: The training objective combined cross-entropy classification loss ℒCE with mean squared error regression loss ℒreg, as shown in Equations 9–11.

+total=+CE+λ·+reg (9)

$$+\mathrm{CE}=-(1/N)\;{\Sigma_{i=1}}^{N}\;{\Sigma_{k=1}}^{K}\;\mathrm{yik}\;\log(\hat{p}\mathrm{ik})$$ (10)

$$+\mathrm{reg}=(1/N)\;{\Sigma_{i=1}}^{N}\;{\left(s_{i}-{\hat{S}}_{i}\right)}^{2}$$ (11)

Where λ = 0.5 balanced the two objectives. This multi-task formulation ensured that the model learned both categorical distinctions between landscape character groups and continuous variations in visual comfort score.

- *Regularization*: To prevent overfitting, several regularization techniques were employed: weight decay (*ω* = 0.05) in the AdamW optimizer, gradient clipping (||∇θ||max = 1.0), and early stopping with patience of 5 epochs based on validation set performance. The batch size was set to B = 16 to balance the gradient estimate quality with the computational constraints.
- *Data partitioning*: The dataset was partitioned into training (75%), validation (10%), and test (15%) subsets using stratified random sampling to ensure balanced representation of all 10 landscape character classes across each split, following established practice for small-to-medium-scale vision classification tasks with multi-class imbalance (Tian and Bai, 2024; Yun, 2025). A fixed random seed (seed = 42) was applied to all stochastic operations to ensure complete reproducibility of the data split and all reported results. All performance metrics reported in Section 3 were computed exclusively on the hold-out test set (*n* = 73 images), which was not accessed at any point during model development, hyperparameter selection, or early stopping.

#### 2.4.3 Attention extraction and visualization protocol

The attention patterns were systematically extracted and interpreted to determine which features drive visual comfort assessments, using the following steps:

- *Attention map aggregation*: For each test image xᵢ, attention weights were extracted from all h = 6 heads across all L = 12 layers, yielding a collection {A1ʰ}1=₁,…,ᴸ; ʰ=₁,…,ʰ of attention matrices. Following established interpretability methods, attention rollout was computed to trace the information flow from the input patches to the classification token, as shown in Equation 12.

$$\bar{A}=\Pi_{l=1}^{L}\;((I+A_{1})/2)$$ (12)

Where *I* is the identity matrix of dimension (N + 1) x (N + 1), A_l_ denotes the attention matrix at layer l averaged across all *h* attention heads. Averaging with the identity matrix (I + A_l_)/2 accounts for residual connections in each transformer layer, ensuring that information flow from earlier layers is preserved. The row of the resulting matrix corresponding to the classification token [cls] is extracted and reshaped from the sequence dimension (*N* = 196) to the spatial grid dimensions H′ x W′, then bilinearly upsampled to the original image resolution (224 × 224) to produce the final spatial attention heatmap.

- *Feature-attention correlation*: To quantify which landscape elements received the most attention, semantic segmentation masks were manually annotated for a subset of images, delineating regions corresponding to vegetation, water, buildings, pathways, and sky. For each semantic category c, the mean attention weight within that region was computed via Equation 13.

$$\bar{\alpha }c=(\frac{1}{|Rc|})\Sigma (i,j)\in Rc\;\bar{A}[i,j]\;)$$ (13)

Where *ℛc* denotes the complete set of spatial pixel coordinates (i, j) belonging to semantic category c (e.g., vegetation, water, buildings, pathway, sky). $\bar{A}[i,j]\;$ is the aggregated attention weight at spatial position (i, j) obtained from Equation 12. The resulting scalar α̅c represents the mean attention weight the model assigns to category c, enabling direct quantitative comparison of model focus across semantic landscape elements.

- *Visualization generation*: Attention maps were overlaid on original images using a heat-map color scheme (viridis), with regions receiving high attention highlighted in warm colors. These visualizations enabled qualitative assessment of whether the model’s focus aligned with design principles, for instance, whether water features and vegetation received greater attention in landscapes with a high visual comfort score.

#### 2.4.4 Evaluation metrics

Unlike purely performance-oriented studies, evaluation prioritized interpretability and alignment with human perception alongside classification accuracy (Yun, 2025).

- *Classification Performance*: Classification performance was assessed using complementary metrics. Accuracy provides an overall measure of correct predictions, as shown in Equation 14. The macro-F1 score offers a balanced evaluation across all landscape character types, as shown in Equation 15.

$$\mathrm{Acc}=(1/N)\;{\Sigma_{i=1}}^{N}\;1[\hat{y}i=\mathrm{yi}]$$ (14)

$$F1\text{macro}=(1/K)\;{\Sigma_{k=1}}^{K}\;(2\cdot P_{k}\cdot R_{k})/(P_{k}+R_{k})$$ (15)

Where P_k_ and R_k_ denote precision and recall for class k, respectively.

- *Regression Performance*: For continuous visual comfort score prediction, standard regression metrics were applied. The mean absolute error (MAE) quantifies the average prediction deviation, as shown in Equation 16. The root mean square error (RMSE) penalizes larger errors more heavily, as shown in Equation 17. The coefficient of determination (R2) measures the proportion of variance explained by the model, as shown in Equation 18.

$$\mathrm{MAE}=(\frac{1}{N})\;{\Sigma_{i=1}}^{N}\;|\mathrm{si}-\hat{s}i|$$ (16)

$$\text{RMSE}=\surd [(\frac{1}{N})\;{\Sigma_{i=1}}^{N}\;{\left(\mathrm{si}-\hat{s}i\right)}^{2}]$$ (17)

$$R^{2}=1-[{\Sigma_{i=1}}^{N}\;{\left(\mathrm{si}-\hat{s}i\right)}^{2}]/[{\Sigma_{i=1}}^{N}\;{\left(\mathrm{si}-\bar{s}\right)}^{2}]$$ (18)

#### 2.4.5 Implementation summary

**Figure 4:**
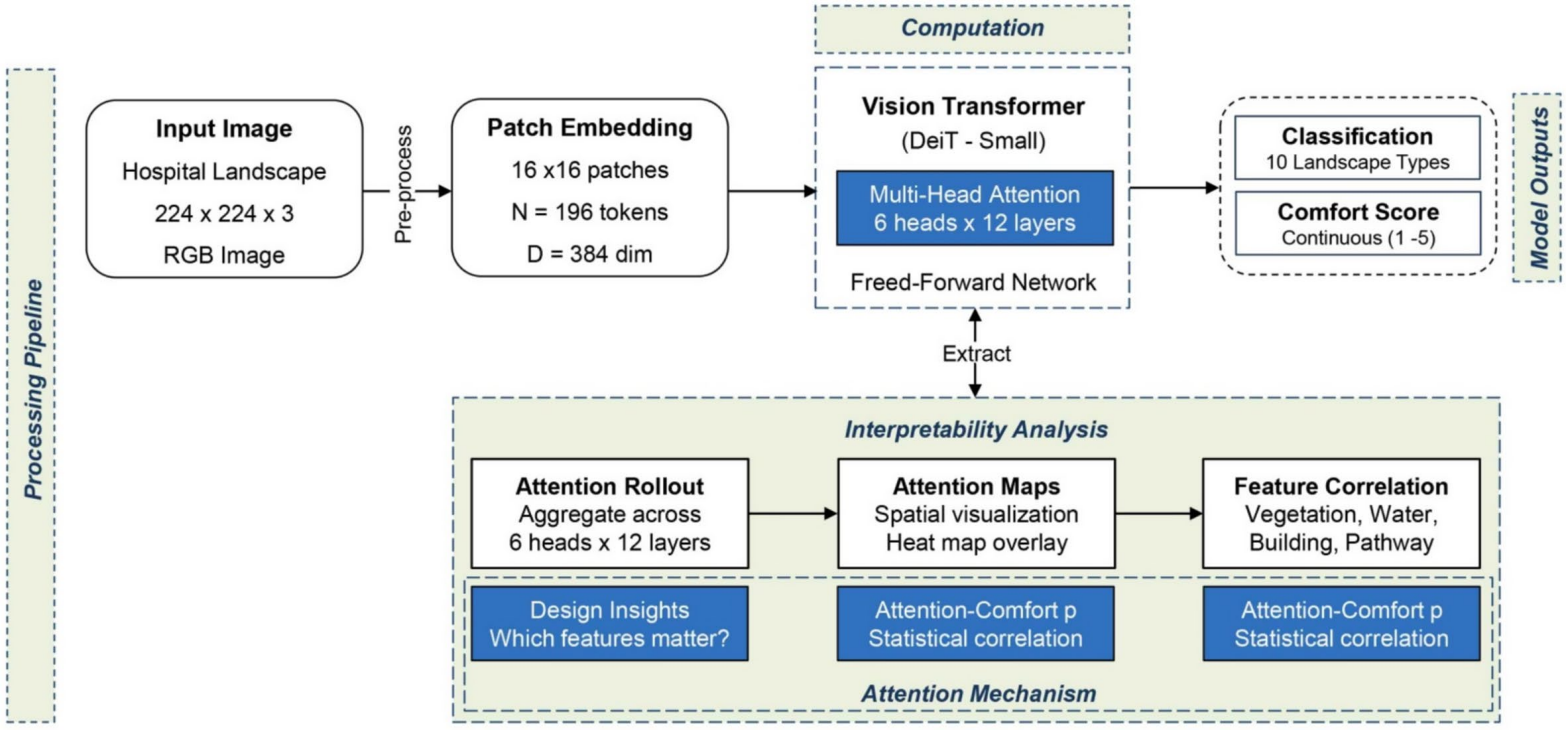
Attention-based visual analysis framework for therapeutic landscape assessment.

The attention-based analysis framework integrated multiple components to extract interpretable insights into therapeutic landscape qualities from computational attention patterns. The process began with standardized hospital landscape images that were pre-processed to ensure consistency while preserving essential visual features. A standard Vision Transformer architecture, DeiT Small, was used to encode spatial relationships through self-attention mechanisms, and the attention weight distributions were then extracted for systematic analysis. Two prediction heads were used to provide categorical landscape-character classification and continuous visual-comfort score estimation, establishing baseline recognition capabilities that enabled attention-perception correlation analysis. The primary analytical contribution was derived from systematic attention extraction and interpretation. Attention maps were aggregated across transformer layers, overlaid on the original images, and correlated with landscape element categories, including vegetation, water, buildings, and pathways to quantify which features received the highest level of model attention. Expert validation by licensed landscape architects ensured that the extracted patterns were consistent with professional design knowledge rather than random correlations. This interpretability-driven methodology produced actionable design insights by revealing which visual elements contribute most to visual comfort, thereby supporting evidence-based landscape planning in hospital environments rather than simply achieving classification performance (see Figure 4 for a framework overview; Supplementary Table C for an implementation pseudocode).

## 3 Results

### 3.1 Respondents’ demographic

Among the 252 respondents who completed the survey, 46.83% were male and 53.17% were female. The age distribution was relatively even, with participants aged 30–39 years forming the largest group (34.13%), whereas those aged 40–49 years represented the smallest group (15.87%). In terms of education, 50.79% of the respondents held an undergraduate degree, and 23.81% held a postgraduate degree. A majority (65.08%) reported being employed. With respect to place of residence, 69.44% lived in urban areas, and 30.56% lived in rural areas. Most respondents also reported prior hospital visits, with 73.41% having stayed in a hospital for more than 24 h. Overall, the participant group consisted mainly of adults of similar age ranges, with relatively high levels of education and prior hospital experience (Table 3).

**Table 3:** Demographic survey.

Category	Sub-category	*N* = 252	Percentages (%)	
Gender	Male	118	46.83	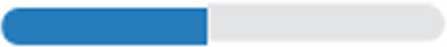
	Female	134	53.17	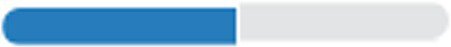
Age	18–29	53	21.03	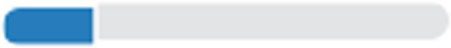
	30–39	86	34.13	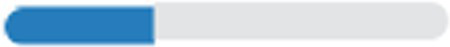
	40–49	40	15.87	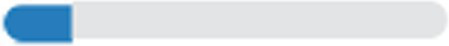
	50–above	73	28.97	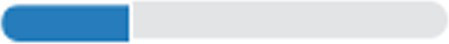
Educational level	High school	64	25.40	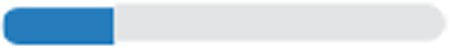
	Undergraduate	128	50.79	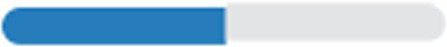
	Postgraduate	60	23.81	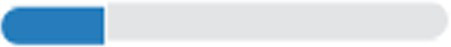
Occupation	Student	39	15.48	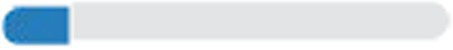
	Employed	164	65.08	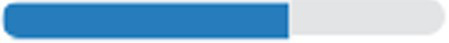
	Unemployed or retired	49	19.44	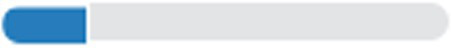
Living area	Urban area	175	69.44	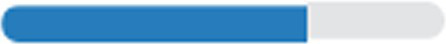
	Rural area	77	30.56	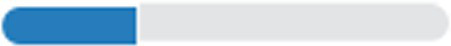
Have you ever visited a hospital?	Yes	251	99.60	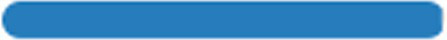
	No	1	00.40	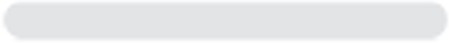
Have you ever stayed in a hospital for more than 24 h?	Yes	185	73.41	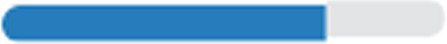
	No	67	26.59	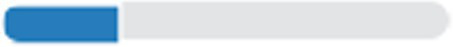
Do you have any background in healthcare?	Yes	78	30.95	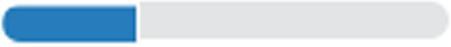
	No	174	69.05	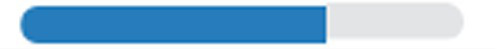

### 3.2 Landscape character scores

#### 3.2.1 Landscape character individual scores

Using descriptive analysis, mean scores were calculated for all the images to establish a baseline understanding of how different scenes enhance visual comfort and perceived therapeutic relaxation in hospital settings. As shown in Tables 4, 5, the two lowest-scoring images were E02 (m = 3.01; enclosed view of green vegetation with two-sided building) and E01 (m = 3.08; enclosed view of green vegetation with two-sided building). These results indicate that images featuring enclosed spaces flanked by buildings or structures are associated with lower visual comfort. In contrast, the highest scores were recorded for images A01 (m = 3.80; broad view of green vegetation) and J02 (m = 3.80; crowded view of green vegetation with water). These findings suggest that open spaces or broad views surrounded by vegetation provide higher visual comfort and that the inclusion of water elements alongside green vegetation further enhances this effect.

**Table 4:** Ranking of individual image mean scores.

NO	Image code	Mean value	NO	Image code	Mean value	NO	Image code	Mean value
1	E02	3.01	11	A02	3.35	21	G01	3.57
2	E01	3.08	12	D02	3.40	22	B01	3.57
3	F01	3.16	13	H02	3.41	23	I02	3.58
4	D01	3.20	14	D03	3.41	24	J03	3.60
5	B02	3.22	15	G03	3.41	25	C02	3.61
6	F02	3.25	16	I01	3.46	26	I03	3.70
7	G02	3.26	17	A03	3.46	27	B03	3.71
8	E03	3.29	18	H01	3.50	28	C03	3.72
9	F03	3.35	19	J01	3.50	29	A01	3.80
10	H03	3.35	20	C01	3.53	30	J02	3.80

**Table 5:** Low-high individual image of visual comfort and therapeutic relaxation.

Visual comfort	Individual scores	
Low visual comfort (LVC)	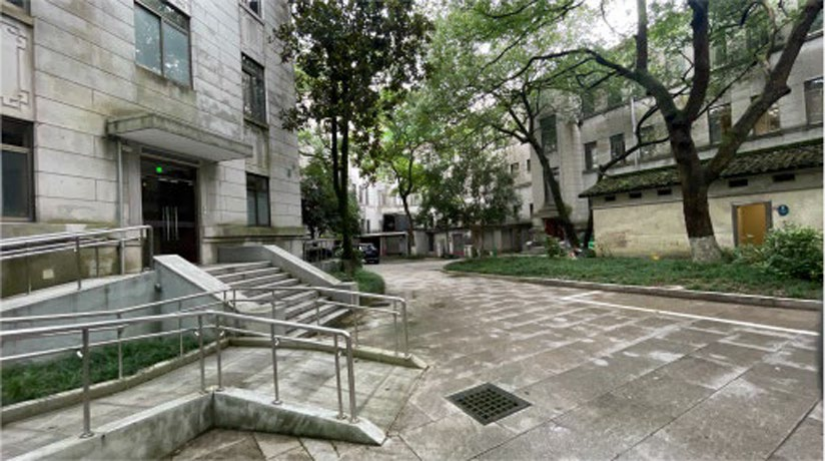	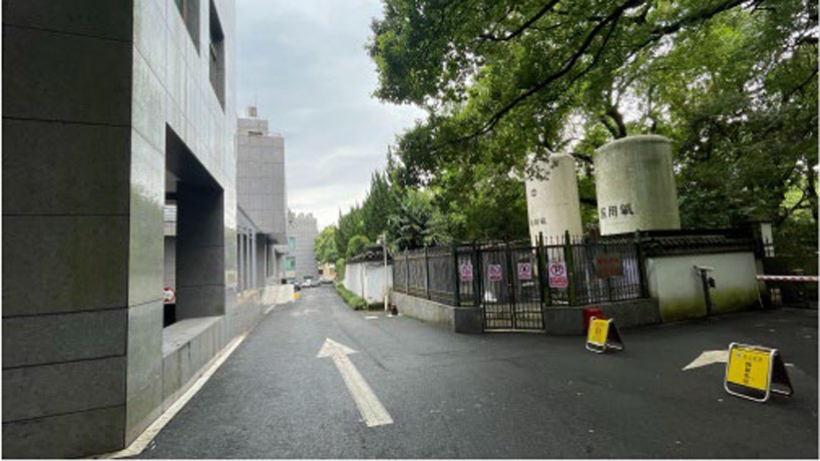
	E02 = 3.01 Enclosed view of green vegetation with two-sided building	E01 = 3.08 Enclosed view of green vegetation with two-sided building
High visual comfort (HVC)	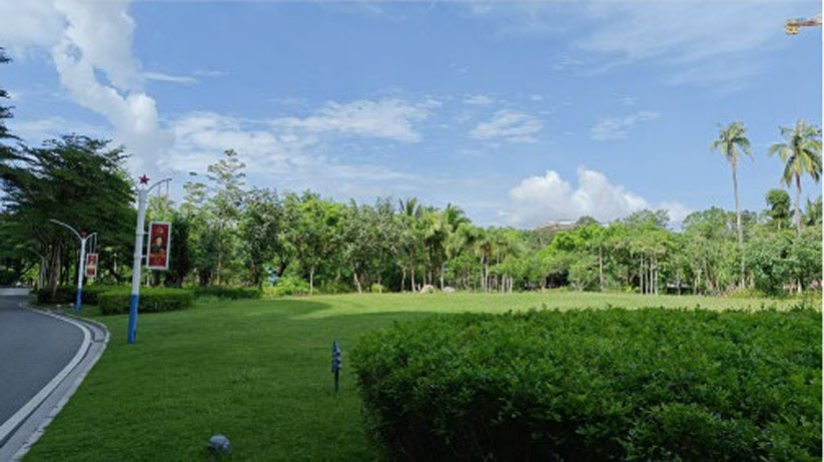	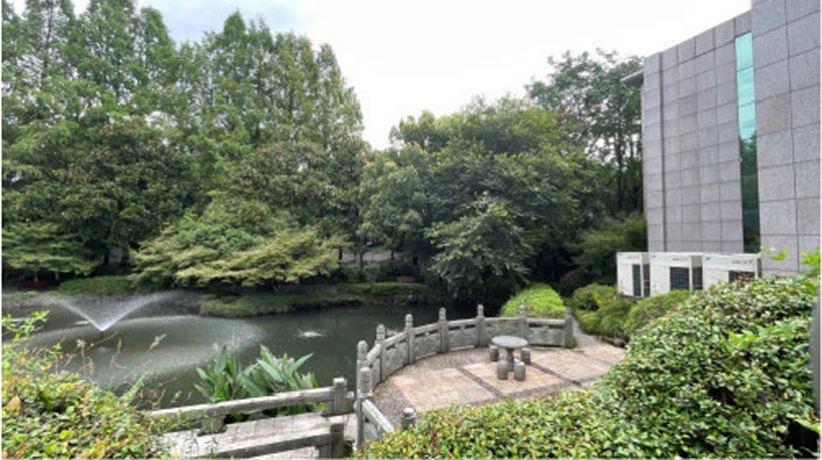
	A01 = 3.80 Broad view of green vegetation	J02 = 3.80 Crowded view of green vegetation with water

#### 3.2.2 Landscape characters group scores

To determine the magnitude of the respondents’ ratings, the mean values for all the images within each landscape character group were averaged. As shown in Table 1, the two least visually comfortable and therapeutic groups were Group E (enclosed view of green vegetation with two-sided buildings, m = 3.13) and Group F (direct view of green vegetation with a building in the background, m = 3.25). These findings are consistent with the single-image results, where participants associated enclosed views, building dominance, and reduced vegetation with lower levels of visual comfort and therapeutic relaxation.

In contrast, the groups with the highest scores were Group C (broad view of green vegetation with building, m = 3.62) and Group J (crowded view of green vegetation with water, m = 3.63). These results reinforce earlier findings: participants perceived broad views of vegetation, as well as water elements combined with greenery, as providing the greatest visual comfort and therapeutic value.

Overall, even the lowest group mean exceeded 3, indicating that all landscape character groups were rated above a moderate level of influence on visual comfort and therapeutic relaxation. Moreover, the results highlight specific reasons for differences in perception: enclosed spaces and building dominance reduced visual comfort, whereas openness, greenery, and water features enhanced it. These human perception scores form a critical link between subjective landscape experience and objective AI training. The gradient from lowest to highest visual comfort scores establishes the value system that the model must learn to replicate, transforming abstract concepts such as “enclosed” and “open” into quantifiable training targets. The consistency of these ratings across 252 respondents provides the statistical foundation necessary for robust machine learning.

### 3.3 AI model performance and learning dynamics

Having established baseline human perception of therapeutic landscape quality, this study further evaluated the capability of deep learning architectures to learn and predict visual comfort and landscape character classification in hospital environments. Three architectures were examined, namely ResNet 50, Vision Transformer ViT, and the proposed DeiT ViT. The findings revealed significant differences in their ability to capture therapeutic landscape qualities that are essential for evidence based healthcare design.

#### 3.3.1 Training dynamics and convergence analysis

**Figure 5:**
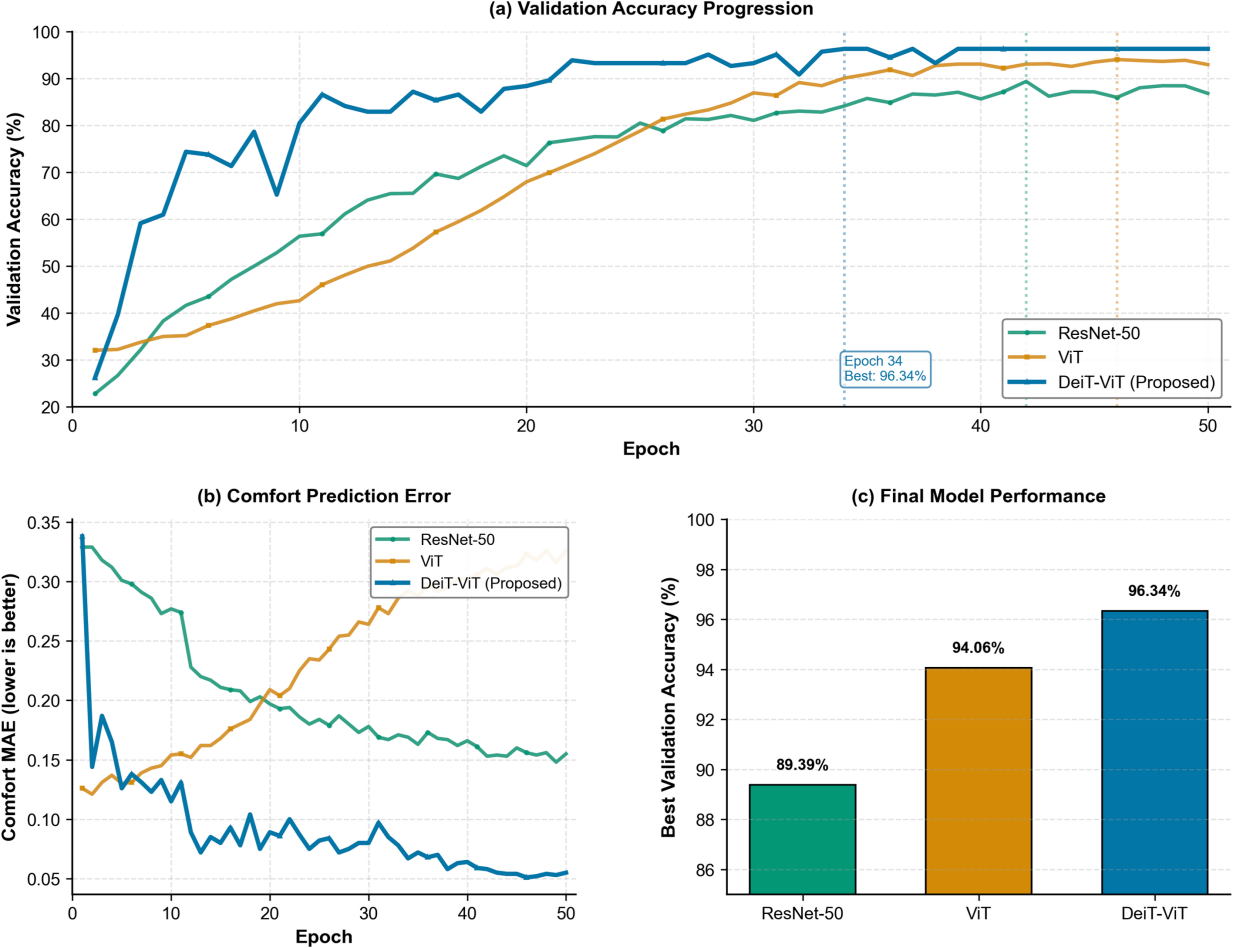
Training dynamics and convergence analysis across 50 epochs. **(a)** Validation accuracy progression; **(b)** Comfort prediction error; **(c)** Final model performance.

Figure 5 presents the comprehensive training dynamics across 50 epochs and reveals fundamental differences in how each architecture learns therapeutic landscape patterns. Figure 5a shows that the DeiT ViT model achieved rapid learning, reaching 75% accuracy by epoch 5 and converging to 96.34% accuracy at epoch 34. This learning trajectory indicates efficient recognition of therapeutic landscape features such as spatial openness, vegetation density, and water presence, which distinguish high comfort landscapes (Groups C and J) from low comfort landscapes (Groups E and F). In contrast, the ViT model plateaued at 94.06% at epoch 46, while the ResNet 50 model plateaued at 89.39% at epoch 42, suggesting limitations in capturing the visual patterns that define therapeutic quality. Figure 5b reveals important differences in visual comfort prediction capability. The DeiT ViT model achieved a mean absolute error of 0.055, representing a 64.5% improvement over the ViT model (0.155) and a 62.8% improvement over the ResNet 50 model (0.148). This result indicates that the DeiT ViT predictions closely approximated human judgments, with an average deviation of only 0.055 points on the five-point Likert scale. Notably, when the ViT model was used, the mean absolute error for visual comfort prediction increased after epoch 10 despite improvements in classification accuracy, suggesting that the model learned categorical distinctions without capturing the nuanced perceptual qualities that determine visual comfort. In other words, it recognized landscape elements but did not fully capture their therapeutic contribution. The consistent reduction in error observed in the DeiT ViT model demonstrates balanced learning of both landscape character recognition and visual comfort assessment. Figure 5c further confirms the performance hierarchy, with DeiT ViT achieving a 6.95 percentage point advantage over ResNet 50 and a 2.28 percentage point advantage over ViT, indicating higher reliability for therapeutic landscape evaluation in clinical environments.

#### 3.3.2 Comprehensive performance evaluation

The proposed DeiT-ViT model exhibited the most expansive coverage, demonstrating particular strength in visual comfort-related dimensions critical for therapeutic landscape character assessment. The balanced profile indicates that the model not only classifies landscape character groups accurately but also reliably predicts visual comfort and perceived therapeutic value, which is essential for practical design applications. The high macro-F1 value suggests balanced recognition across all landscape characters, including low-comfort minority groups (Group E).

Error scores (MAE, RMSE, R^2^) were used to evaluate successful learning of the relationship between visual elements and visual comfort, explaining why broad views with vegetation and water (Groups C and J) elicit higher visual comfort than enclosed building-dominated views (Groups E and F). The ViT model predicted landscape character group reasonably well but did not predict visual comfort well, reinforcing that standard transformers may optimize categorical discrimination without capturing visual qualities.

**Figure 6:**
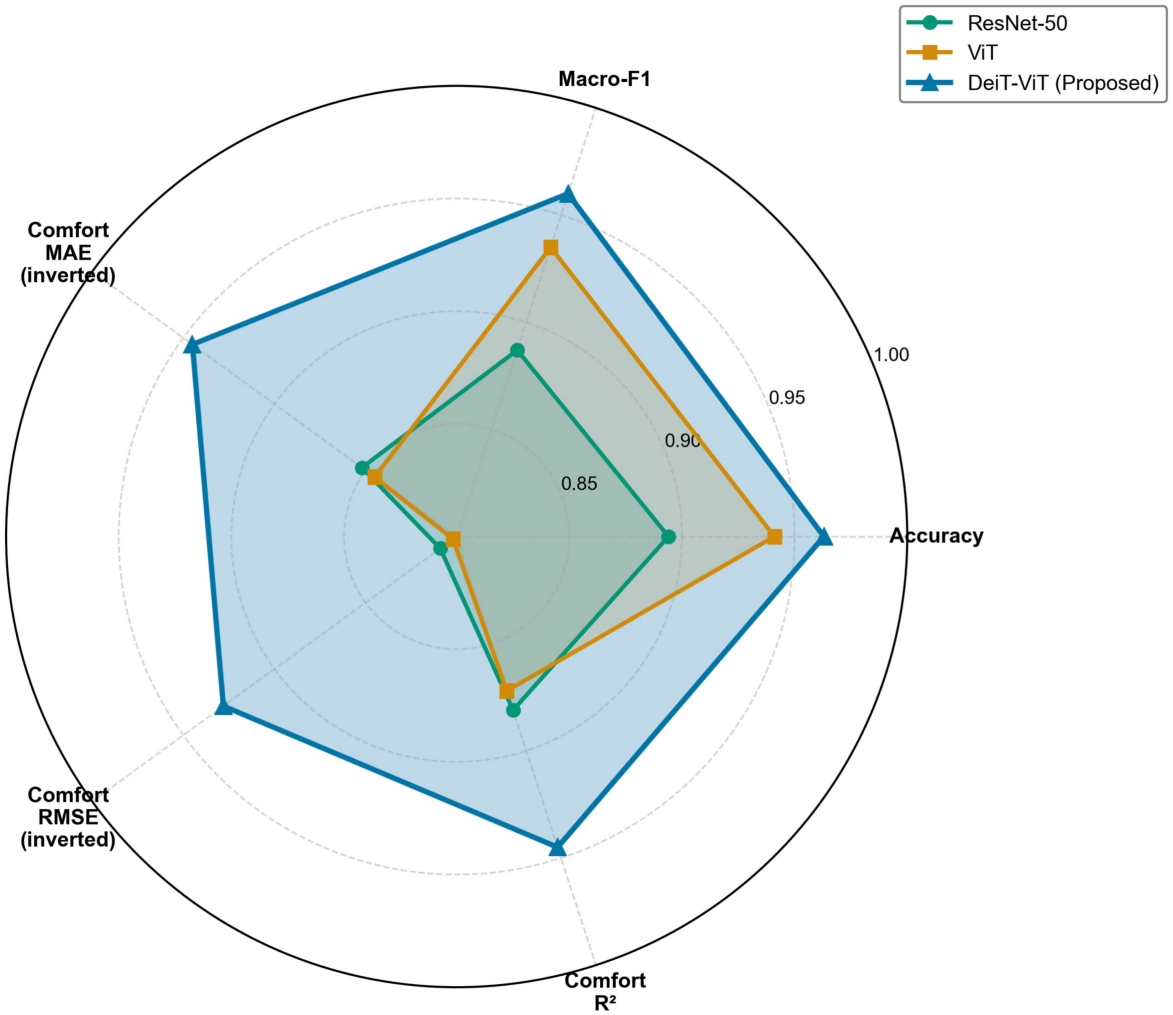
Comprehensive performance evaluation across five metrics (accuracy, macro-F1 score, inverted comfort MAE, inverted comfort RMSE, and comfort R^2^).

The ResNet-50 model had the lowest overall performance, indicating fundamental architectural constraints in learning both landscape character distinctions and visual comfort gradients. Figure 6 provides a comprehensive visualization of performance across five metrics (accuracy, macro-F1, comfort MAE inverted, comfort RMSE inverted, and comfort R^2^).

The DeiT ViT model achieved an accuracy of 96.34% and a mean absolute error of 0.055, enabling predictions that deviated from human judgments by only 0.055 points, which represents a level of accuracy that is critical for evidence-based landscape design decisions. The model converged at epoch 34, representing 26% faster learning than the ViT model (epoch 46) and 19% faster than the ResNet-50 model (epoch 42), demonstrating efficient therapeutic pattern acquisition. While the DeiT-ViT and ViT models share 86.6 M parameters (3.4 × larger than the ResNet-50 model’s 25.6 M), the DeiT-ViT model had a marginal 0.8-h training increase (13.1 vs. 12.3 h), yielding substantial gains: +2.28 percentage point accuracy and −64.5% comfort prediction error. These metrics establish the DeiT-ViT model as the optimal model for therapeutic landscape assessment, combining superior predictive accuracy with efficient convergence and reasonable computational requirements. Table 6 provides a quantitative comparison across the five indicators.

**Table 6:** Quantitative comparison across five indicators.

Model	Accuracy (%)	Comfort MAE	Convergence epoch	Parameters (M)	Training time (h)
ResNet-50	89.39	0.148	42	25.6	8.5
ViT	94.06	0.155	46	86.6	12.3
DeiT-ViT (Proposed)	96.34	0.055	34	86.6	13.1

#### 3.3.3 Mechanistic performance analysis

**Figure 7:**
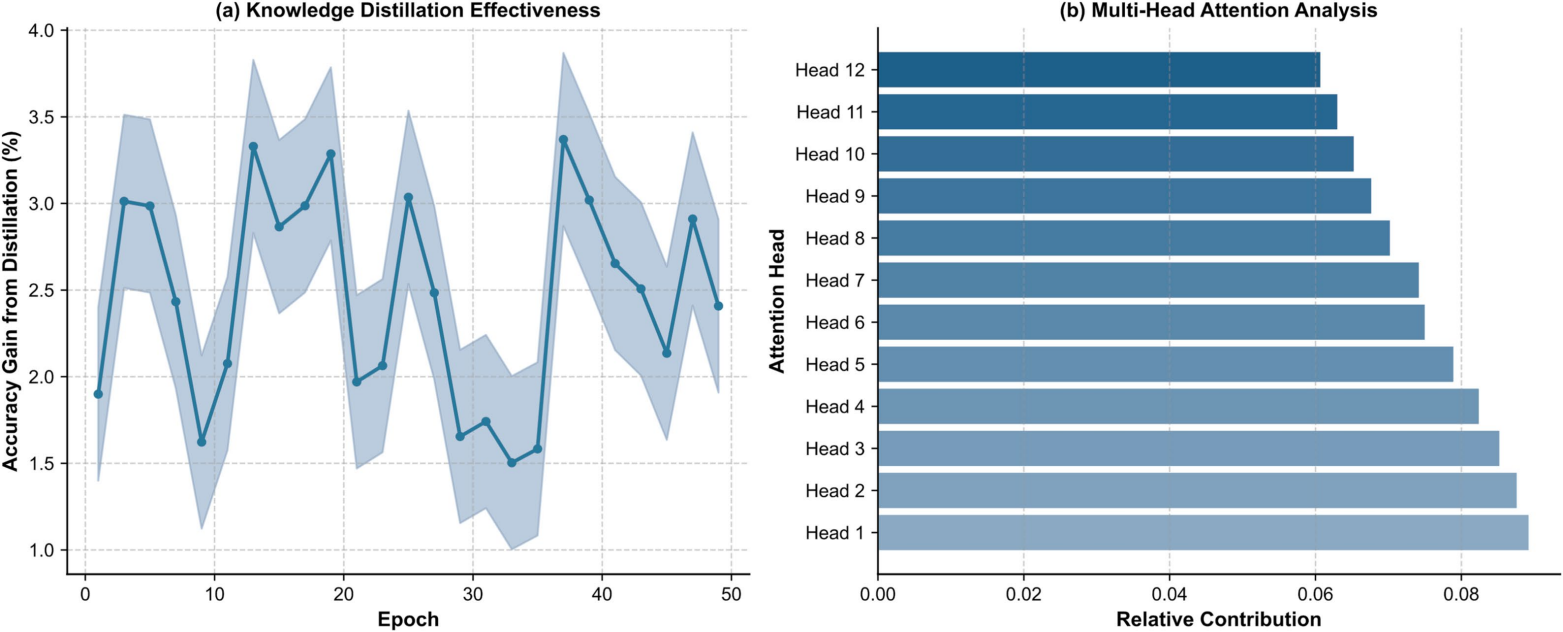
Mechanistic performance analysis of the DeiT-ViT model: **(a)** knowledge distillation and **(b)** multi-head attention investigation.

Various architectural mechanisms underly the DeiT-ViT model’s therapeutic landscape assessment capability. Figure 7a shows that knowledge distillation contributes consistent 1.5–3.5% accuracy improvements throughout training, with peaks reaching 3.0–3.5% at epochs 3, 13, 18, and 37. This sustained benefit indicates that the teacher–student paradigm provides continuous guidance for learning subtle therapeutic landscape qualities, such as optimal vegetation–openness balance or water feature presence across different spatial configurations beyond what labeled data alone provide.

The persistent contribution stabilizing at 2.4% by epoch 50 confirms that knowledge transfer remains valuable throughout learning rather than diminishing as the student develops independent capacity, representing a critical architectural advantage for capturing complex therapeutic landscape patterns. This balanced profile indicates diverse attention mechanisms rather than concentrated head reliance, suggesting that different heads specialize in complementary therapeutic attributes: vegetation characteristics, water features, spatial enclosure, and built–natural element relationships.

The uniform distribution aligns with landscape architecture theory, emphasizing that therapeutic quality emerges from holistic composition rather than isolated elements. This architecture enables robust evaluation across landscape types, distinguishing different therapeutic mechanisms, for example, broad vegetation views (Group A) providing comfort from openness and nature exposure, and crowded water views (Group J) providing a similar level of comfort through restorative water qualities despite reduced openness.

The balanced attention supports comprehensive therapeutic assessment, contributing to the superior landscape character classification accuracy and visual comfort prediction demonstrated in prior analyses. Figure 7b shows multi-head attention contributions were relatively evenly distributed (range: 0.075–0.085), with Heads 5–7 exhibiting marginally higher values (~0.085) and Head 12 the lowest (~0.075).

Collectively, these results establish that the superiority of the DeiT-ViT model de-rived from fundamental architectural advantages: knowledge distillation enabled 19–26% faster convergence with a 2–7 percentage-point higher accuracy and 62–65% lower error for visual comfort prediction, and balanced multi-head attention integrating diverse therapeutic visual features. These findings demonstrate that the DeiT-ViT model is a computationally efficient and perceptually accurate tool for AI-driven hospital landscape assessment, with practical applications in evidence-based design, systematic evaluation of existing therapeutic spaces, and optimization of landscape configurations enhancing patient recovery and wellbeing.

## 4 Discussion

This study presents several important findings on human visual perception in hospital landscapes. A questionnaire with 252 respondents provided perception scores for different landscape characters, assessing visual comfort and therapeutic relaxation in Chinese hospital settings. The combined results showed that even the lowest-scoring landscape character groups or single images exceeded m = 3.00, indicating that all landscapes had at least a moderate positive influence on visual comfort and perceived therapeutic relaxation. This can be attributed to the presence of green vegetation in all images, suggesting that vegetation consistently enhances visual comfort and relaxation. These findings align with those of Olszewska-Guizzo et al. (2022), who demonstrated that therapeutic gardens promote positive emotions, relaxation, and improved brain activity, thereby accelerating patient recovery. They are also supported by the Attention Restoration Theory, which highlights the psychological benefits of natural scenes (Kang and Kim, 2019).

A further methodological consideration concerns the composition of the survey sample and the potential for subjective variance across respondent health status. The 252 survey participants were recruited from the general public and were not stratified by clinical condition, health status, or current hospitalization. Consequently, the visual comfort scores reflect the landscape preferences of predominantly healthy adults rather than patients who are the primary end-users of hospital therapeutic landscapes. Research in environmental psychology and healthcare design indicates that clinical populations may evaluate outdoor landscape stimuli differently from healthy individuals, owing to altered attentional capacity associated with pain, fatigue, or psychological distress, as well as modified restorative needs during recovery (Bernhardt et al., 2022; Simonsen and Duff, 2020). In particular, hospitalized patients may place greater therapeutic value on enclosed, shaded, and private spaces that provide a sense of security and refuge, in contrast to the open broad views and water features most preferred by the healthy respondents in the present study. This divergence implies that the visual comfort scores used to train and validate the DeiT-ViT model may not fully capture patient-specific perceptual states, potentially limiting the direct transferability of the model’s predictions to clinical design decisions. Future studies should recruit stratified samples including currently hospitalized patients, outpatients at different stages of recovery, and healthy control groups, to determine whether group-level differences in visual comfort ratings are statistically significant. If substantial perceptual differences are confirmed, condition-specific prediction models or adaptive scoring systems may be warranted to ensure that AI-driven therapeutic landscape assessments accurately reflect the preferences of the populations they are designed to serve.

Moreover, the results revealed that views enclosed by buildings on two sides, or direct views dominated by buildings with limited vegetation, were associated with de-creased comfort and relaxation. This outcome is consistent with Weng et al. (2022), who reported that enclosed building forms cause discomfort for pedestrians because of the sense of spatial confinement. However, the findings differ from those of Izadyar et al. (2023), who argued that surrounding buildings can enhance natural ventilation and thermal comfort, both of which are essential to therapeutic environments. In contrast, broad views of green vegetation and the presence of water elements alongside vegetation were associated with higher levels of visual comfort and relaxation. This preference for openness and water features may reflect traditional Chinese garden principles of borrowed scenery as well as the cultural significance of water as a therapeutic element. These findings are consistent with Gómez et al. (2018), who reported that the design of natural elements, particularly diverse plants and water features, reduces stress and enhances comfort in urban settings. Similarly, Mundher et al. (2023b) demonstrated that openness and water in natural environments are among the most important factors shaping aesthetic experience, directly influencing visual comfort and therapeutic relaxation.

The strong performance of the DeiT-ViT model demonstrates that therapeutic landscape qualities, often regarded as subjective and culturally specific, can be systematically learned by AI systems. With an accuracy of 96.34% and an MAE of 0.055 for visual comfort prediction, the model successfully achieved dual objectives: accurate landscape character classification and precise visual comfort quantification that closely approximates human judgments. This performance suggests that visual patterns contributing to visual comfort exhibit learnable regularities within the Chinese hospital context, enabling AI-driven evidence-based landscape design. The DeiT-ViT model’s multi-head attention mechanisms consistently highlight design elements recognized in landscape architecture literature as central to therapeutic experiences: spatial enclosure degree, water feature presence, vegetation density, and natural–built element relationships. This alignment between computational attention and landscape architecture validates both the AI-driven approach and existing therapeutic landscape principles. For practicing landscape architects, the DeiT-ViT model offers several practical applications. It enables rapid evaluation of existing hospital landscapes to identify areas requiring improvement, with lower-scoring spaces prioritized for renovation. It informs design phases, allowing architects to test different configurations of vegetation, water features, and architectural elements to optimize visual comfort. The model’s empirical validation, with a 64.5% lower MAE for visual comfort prediction over standard transformers, indicates that this is a valuable tool for justifying landscape investments to hospital administrators through quantifiable therapeutic impact assessments.

## 5 Limitations and future studies

Several limitations warrant consideration. This study was conducted at the land-scape level within Chinese hospital settings; as such, the dataset’s scope and representativeness may restrict the generalizability of the findings to other settings, contexts, or landscape typologies not adequately represented in the training data. Future studies could extend this research to different contexts or countries, enabling meaningful cross-comparisons. The current dataset of 488 images, though carefully curated, may not capture the full diversity of hospital landscapes across China. Expanding future datasets to more than 500 images would help encompass a broader range of environmental conditions and typologies. Moreover, the reliance on ground-level imagery (1.70 meters, at eye level) captures only the human-scale perspective, excluding features that might be visible from alternative viewpoints. Incorporating drone-based aerial imagery could significantly enhance the model’s ability to interpret spatial context and landscape composition. Another limitation is the subjectivity of perceptual labels: human evaluations of comfort and relaxation inherently vary, introducing potential noise that may affect model learning and assessment. In addition, this study did not distinguish between responses from healthy individuals and hospital patients, whose perceptions of therapeutic landscapes may differ due to variations in physical condition, psychological state, and environmental needs; future research should compare these groups to better understand perception differences in healthcare environments. Additional sources of variability, such as weather conditions, seasonal changes, and demographic characteristics of respondents, may also influence ratings of therapeutic quality. Finally, external validity remains a critical concern. Future research should test model performance on independent datasets collected in different geographic regions and cultural contexts.

## 6 Conclusion

This study highlights the close relationship between landscape design and human visual perception in hospital environments. The findings show that all landscape elements examined achieved above-average levels of visual comfort and perceived therapeutic relaxation, with greenery emerging as the most influential factor. Landscapes rich in vegetation and complemented by water features provided the highest levels of visual comfort, whereas those enclosed by buildings or with limited greenery reduced visual comfort. In addition to human perception, the study demonstrated that AI can systematically capture and interpret these qualities. The DeiT-ViT model achieved 96.34% classification accuracy and an MAE of 0.055 for visual comfort prediction, successfully fulfilling the dual objectives of accurate landscape character recognition and precise visual comfort quantification. Compared with the baseline architectures, the model’s knowledge distillation mechanism enabled 19–26% faster convergence, whereas the balanced multi-head attention facilitated the comprehensive integration of diverse landscape features. These results suggest that visual patterns contributing to visual comfort exhibit consistent, computationally learnable regularities across individuals. This convergence between design theory and AI-based evaluation, validated through a 64.5% lower MAE for visual comfort prediction over standard transformers, underscores the potential of combining human-centered insights with advanced computational tools.

Hospital landscapes can be assessed and improved via AI-driven tools that support both evaluation and design. Patients stand to benefit the most, with more therapeutic environments that promote comfort and recovery. Hospital visitors and staff also gain from landscapes that reduce stress and support mental wellbeing. For landscape architects and designers, the findings offer evidence-based guidance for creating therapeutic spaces, whereas administrators and policymakers can draw on quantifiable therapeutic impact assessments to inform investment and planning decisions. Finally, researchers benefit from the study’s contribution to demonstrating how AI can advance evidence-based design in healthcare settings through interpretable attention mechanisms that align with established therapeutic landscape principles.

## Data Availability

The original contributions presented in the study are included in the article/Supplementary material, further inquiries can be directed to the corresponding author.
