# Supplementary material for: Data-efficient image transformer for landscape character classification and visual comfort prediction in Chinese hospitals
Source: Front Artif Intell. 2026 May 8;9:1733709. doi: 10.3389/frai.2026.1733709 (PMC13194462; doi:10.3389/frai.2026.1733709)
Supplement: Supplementary file 1 [file Data_Sheet_1.PDF]

Supplementary Material

Table A. Image and Code Display for Landscape Character

A - Broad view of green vegetation

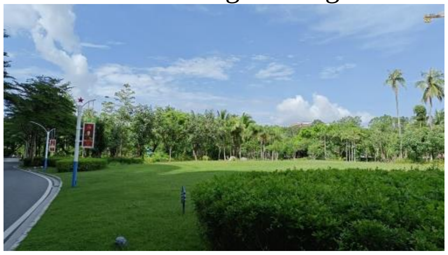
A01

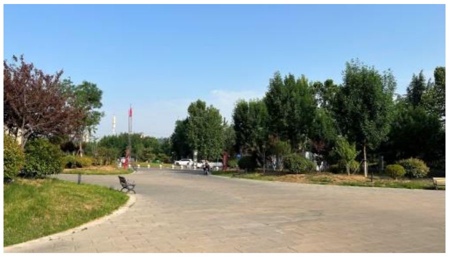
A02

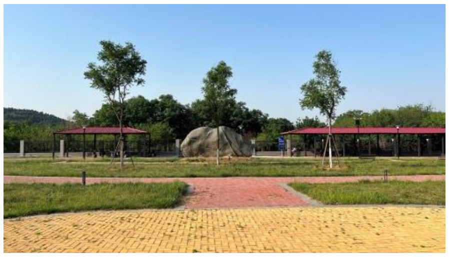
A03

B - Crowded view of green vegetation

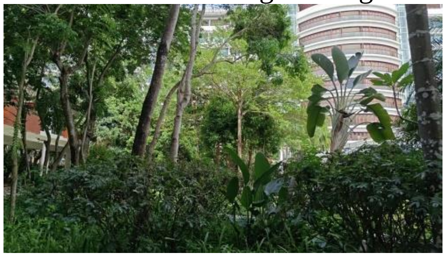
B01

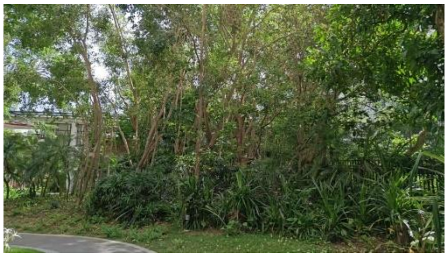
B02

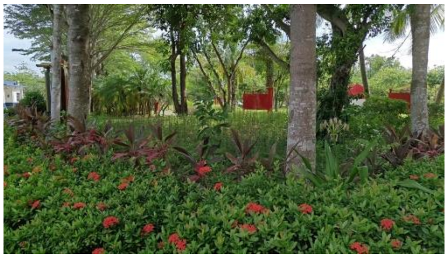
B03

C - Broad view of green vegetation with building

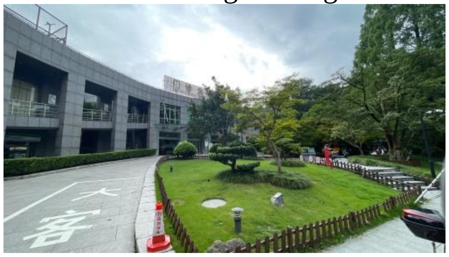
C01

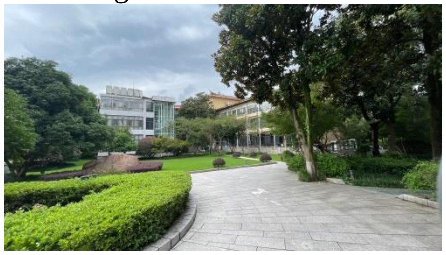
C02

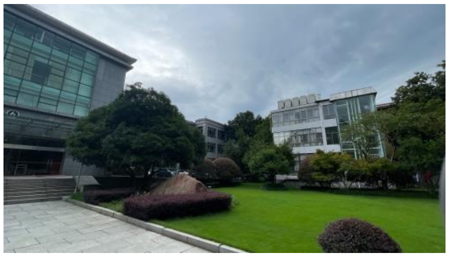
C03

D - Enclosed view of green vegetation with one-sided building

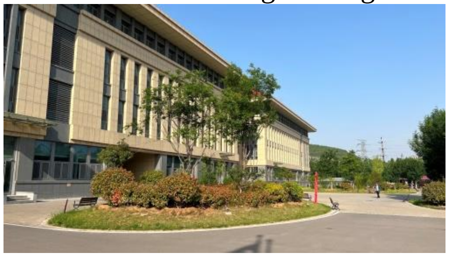
D01

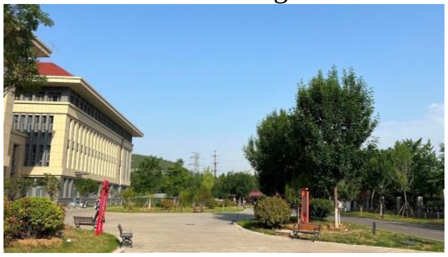
D02

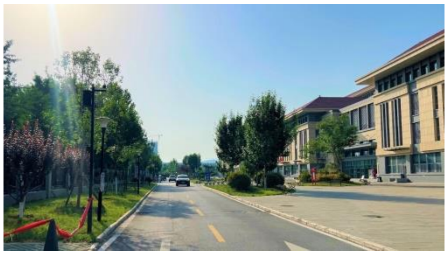
D03

E - Enclosed view of green vegetation with two-sided building

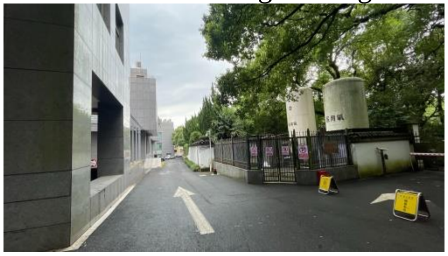
E01

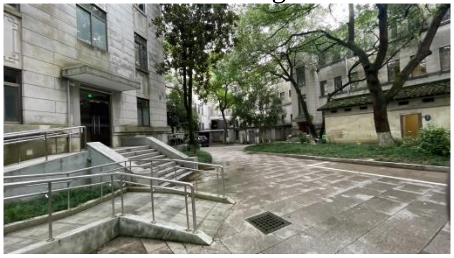
E02

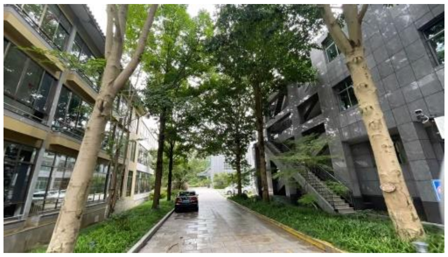
E03

F - Direct view of green vegetation with background building

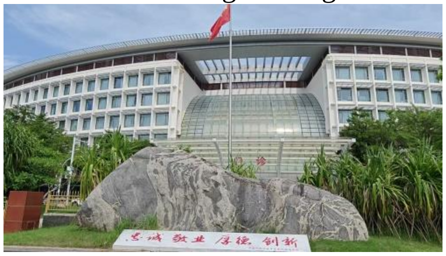

F01

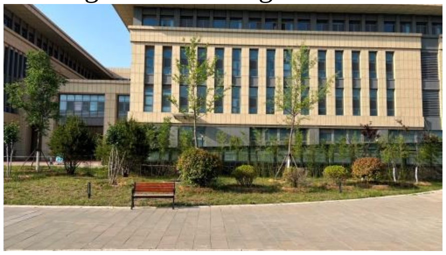

F02

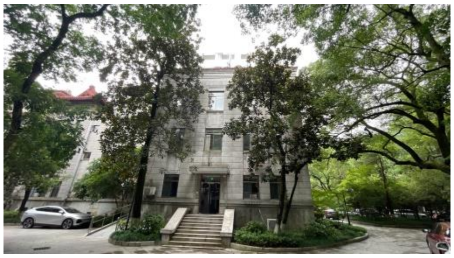

F03

G - Direct view of green vegetation with Infrastructure

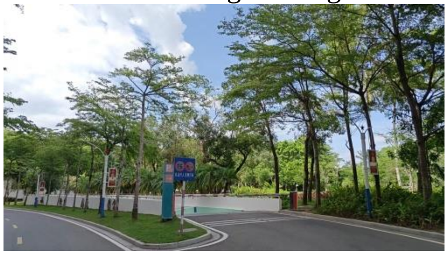

G01

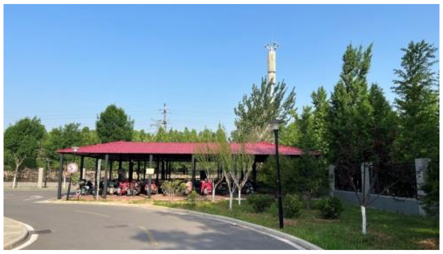

G02

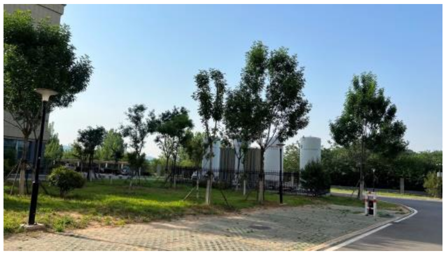

G03

H - Crowded view of green vegetation with Infrastructure

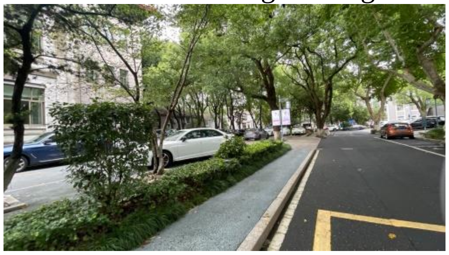

H01

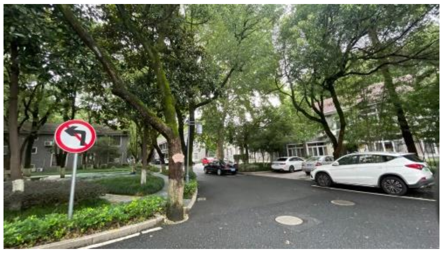

H02

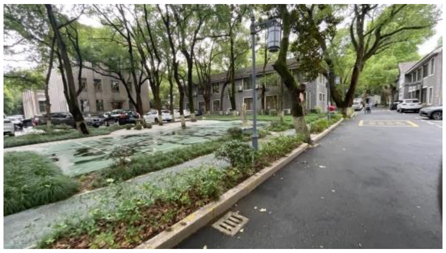

H03

I - Enclosed view of green vegetation with pathway

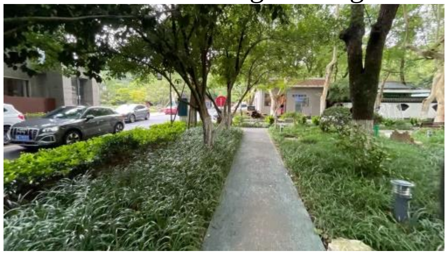

I01

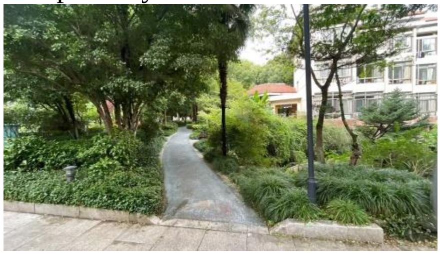

I02

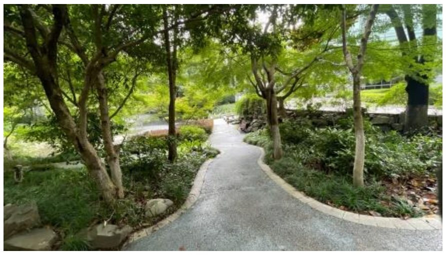

I03

J - Crowded view of green vegetation with water

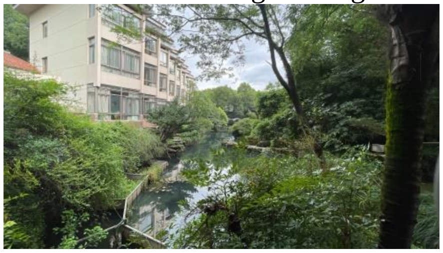

J01

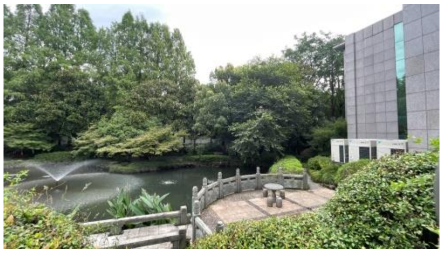

J02

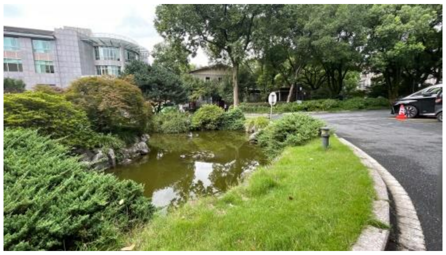

J03

Table B. **Mathematical Notation**

Symbol	Value / Range	Description
	Scalars, Dimensions, and Hyper-parameters	
B	16	Training batch size
D	384	Hidden dimension of transformer patch embedding space
d_k	$D/h = 64$	Dimensionality per attention head
h	6	Number of parallel attention heads
K	10	Number of landscape character classes
L	12	Number of transformer encoder layers
N	488 / 196	Total images in dataset (488); image patch tokens per image (196)
P	16 px	Patch size ($P \times P$ pixels per non-overlapping patch)
λ	0.5	Loss weighting coefficient balancing classification and regression losses
η_1	5×10^{-4}	Stage 1 (feature extraction) learning rate
η_2	5×10^{-5}	Stage 2 (fine-tuning) initial learning rate
η_{min}	1×10^{-6}	Minimum learning rate at end of cosine annealing schedule
η_{max}	$\eta_2 = 5 \times 10^{-5}$	Maximum learning rate at start of cosine annealing schedule
ω	0.05	Weight decay coefficient in AdamW optimiser
T_1	20 epochs	Number of epochs in Stage 1 (heads-only training)
T_2	30 epochs	Number of epochs in Stage 2 (full fine-tuning)
	Input, Output, and Dataset Variables	
x_i	$\in \mathbb{R}^{(H\times W\times 3)}$	Input RGB image i
g_i	$\in \{A, B, \ldots, J\}$	Landscape character group label for image i
s_i	$\in [1, 5]$	Ground-truth visual comfort score for image i (Likert scale)
$\hat{s}$	$\in [1, 5]$	Predicted visual comfort score output by regression head
$\bar{s}$	—	Mean visual comfort score across the test set (used in R^2)
p	$\in \mathbb{R}^K$	Predicted class probability vector from classification head
	Architecture Matrices and Vectors	
E	$\in \mathbb{R}^{((P^2\cdot 3)\times D)}$	Learnable patch projection matrix
E_{pos}	$\in \mathbb{R}^{((N+1)\times D)}$	Positional encoding matrix added to patch embeddings
z_0	$\in \mathbb{R}^{((N+1)\times D)}$	Initial token sequence (CLS token + projected patches + positional encodings)
$z_{cls}^{(L)}$	$\in \mathbb{R}^D$	Final CLS token representation from last transformer layer (used for prediction)
Q	$\in \mathbb{R}^{(N\times d_k)}$	Query projection matrix in scaled dot-product attention
K	$\in \mathbb{R}^{(N\times d_k)}$	Key projection matrix in scaled dot-product attention
V	$\in \mathbb{R}^{(N\times d_k)}$	Value projection matrix in scaled dot-product attention
W_c	$\in \mathbb{R}^{(D\times K)}$	Learnable weight matrix of classification head
W_r	$\in \mathbb{R}^{(D\times 1)}$	Learnable weight matrix of regression head
b_c	$\in \mathbb{R}^K$	Bias vector of classification head
b_r	$\in \mathbb{R}$	Bias scalar of regression head
I	$\in \mathbb{R}^{((N+1)\times(N+1))}$	Identity matrix (used in attention rollout, Equation 12)
A_l^h	$\in \mathbb{R}^{(N\times N)}$	Attention weight matrix at layer l, head h
$\bar{A}$	$\in \mathbb{R}^{(1\times N)}$	Aggregated attention map (attention rollout over all layers and heads)
	Attention Mechanisms and Interpretability	

$\bar{\alpha}$_c	$\in \mathbb{R}$	Mean attention weight assigned to semantic category c
$\mathscr{R}$_c	—	Set of pixel coordinates (i,j) belonging to semantic category c
$\mathscr{L}$_CE	—	Cross-entropy classification loss
$\mathscr{L}_{reg}$	—	Mean squared error regression loss for visual comfort prediction
	Loss Functions and Activation	
$\mathscr{L}$_total	$\mathscr{L}$_CE + λ·$\mathscr{L}$_reg	Combined multi-task training loss
σ(·)	—	Sigmoid activation function — maps linear output to (0,1)

Table C. Attention-Based Visual Analysis for Therapeutic Landscape Assessment

Algorithm 1 Attention-Based Visual Analysis for Therapeutic Landscape Assessment

Require: $\mathcal{D} = \{(x_i, g_i, s_i)\}_{i=1}^{N}$; $K{=}10$, $B{=}16$, $T_1{=}20$, $T_2{=}30$, $\eta_1{=}5\times10^{-4}$, $\eta_2=5\times10^{-5}$, $\lambda=0.5$

Ensure: M_*, attention maps $\{H_i\}$, correlations $\{\rho_c\}$, metrics

Set seed= 42; split D → D_{train}(75%),D_{val}(10%),D_{test}(15%) stratified

Load DeiT-Small (ImageNet; L=12, h=6, D=384); add heads $W_c \in \mathbb{R}^{K\times D}$, $W_r \in \mathbb{R}^{1\times D}$

Stage 1: Freeze backbone, train heads (T1 epochs)

for e = 1 to T_1 do

for (X,G,S) ∈ D_{train}, size B do

Z ← [CLS;PatchEmbed(T (X))]+E_{pos}; run L transformer layers

$\hat{p}$ ← SoftMax ($W_c z_{cls}+b_c$); $\hat{s}$ ← $4\sigma(W_r z_{cls}+b_r)+1$; L ← CE ($\hat{p}$,G) Update heads only via AdamW(η_1)

Validate; early stop (patience= 5)

Stage 2: Fine-tune all params (T2 epochs)

for e = 1 to T_2 do

for (X,G,S) do

Forward pass; L ← CE ($\hat{p}$,G)+λMSE($\hat{s}$,S); clip $\|\nabla\|\le1$; update all θ via AdamW(η_t)

Save M_* by best Macro-F1

Attention Extraction (Primary Contribution)

for x_i ∈ D_{test} do

Run forward; collect A_l^h ($l{=}1..L$, $h{=}1..6$); get predictions $\hat{y}_i$,$\hat{s}_i$

Rollout: $\bar{A} \leftarrow I$; for $l{=}1..L$: $\bar{A} \leftarrow \bar{A}\cdot\frac{I+\frac{1}{6}\sum_h A_l^h}{2}$

Extract a ← $\bar{A}$[0,1:197]; reshape/upsample to $H_i \in \mathbb{R}^{224\times224}$

Feature Correlation

Annotate masks $M_{i,c}$ for C={veg, water, buildings, paths, sky}

for c ∈ C do

$\alpha_{i,c}$ ← mean{H_i[x,y] | $M_{i,c}$[x,y]=1} for all annotated i

ρ_c ← Spearman({$\alpha_{i,c}$},{s_i})

Attention Consistency

for class g ∈ {A..J} do

Binarize H_i at median for all i with $\hat{y}_i$=g; Consistency_g ← Mean (pairwise IoU)

Evaluate

Acc, Macro-F1 (classification); MAE, RMSE, R^2 (regression); report {ρ_c}, {Consistency_g}

return M_*, metrics, {H_i}, {ρ_c}
